# Insect floral visitors of thermo-Mediterranean shrubland maquis (Ajaccio, Corsica, France)

**DOI:** 10.3897/BDJ.12.e118614

**Published:** 2024-04-25

**Authors:** Pierre-Yves Maestracci, Laurent Plume, Marc Gibernau

**Affiliations:** 1 CNRS – University of Corsica - Laboratory Sciences for the Environment (UMR 6134 SPE), Natural Resources Project, Ajaccio, France CNRS – University of Corsica - Laboratory Sciences for the Environment (UMR 6134 SPE), Natural Resources Project Ajaccio France; 2 ENGIE-Lab-CRIGEN, Stains, France ENGIE-Lab-CRIGEN Stains France; 3 University Paris-Panthéon-Assas, Laboratory Management Research (Largepa), Paris, France University Paris-Panthéon-Assas, Laboratory Management Research (Largepa) Paris France

**Keywords:** insects, pollinators, Coleoptera, Hymenoptera, Diptera, Lepidoptera

## Abstract

**Background:**

The Mediterranean Region represents a biodiversity hotspot with a high rate of endemism. In its western part, Corsica Island is notable in terms of biodiversity due to its large surface and its large range of habitats from seaside to alpine biotopes. Amongst diverse groups, insects, notably the main orders of pollinators composed of Coleoptera, Hymenoptera, Diptera and Lepidoptera, represent a good part of the insular richness.

**New information:**

Our sampling effort focused on the insects from these four orders visiting flowers in a characteristic thermo-Mediterranean vegetation. Our database is an insight into the Corsican floral visitor biodiversity from three sites separated by a few kilometres in the region of Ajaccio during 13 months over two successive years. In total, 4012 specimens were sampled and 252 species or morpho-species identified from 133 genera and 47 families. Beetles were by far the most abundant order representing about 54% of the sampled specimens. The most diverse order was the Hymenoptera representing 39% of the species. Our continuous survey showed that these orders are temporally dynamic both between years and between seasons in terms of abundance and diversity.

## Introduction

Most of the 400,000 flowering plants are pollinated by animals and a recent global estimate suggests that 87.5% of angiosperms rely on invertebrates or vertebrates in this way (Ollerton 2017). Actually, there are approximately 350,000 known species of pollinators and 98.4% of them are insects from the four orders Lepidoptera, Coleoptera, Hymenoptera and Diptera (Ollerton 2017). In the context of global change and preservation of biodiversity, listing species diversity is important, but understanding of how an ecosystem functions is a key component to conserve ecosystems (Weisser and Siemann 2008). Plant–pollinator interactions represent a major ecosystem function not only for conservation biology, but also for the evolution of many terrestrial ecosystems as species diversity of pollinators is crucial for plant reproduction (Layek et al. 2022). Pollinator diversity is not evenly distributed in space, it follows the expected pattern of increasing species richness with latitude, the Tropics having more pollinators and richer floras (Ollerton 2017). However, it has long been known that the diversity of bees, one of the major groups of pollinators, peaks not in the Tropics, but rather in dry, subtropical, Mediterranean-type communities (Michener 2007, Ollerton 2017).

In Europe, the species richness is explained by the diversity of landscapes, their structure and the weather seasonality (Ollerton 2017). In mainland France, a country with a large diversity of landscapes, it is estimated that more than 20,000 insect species feed in flowers (I.P.B.E.S. 2016, Reverté 2023) with a highest richness in the south, the Mediterranean Region, a biodiversity hotspot (Mittermeier et al. 2004).

In Corsica, significant work on insect diversity has been carried out in recent years, including the MNHN “Planète revisitée” expeditions (Ichter et al. 2021, Ichter et al. 2022) and works of the Territory with its reference organisation: the OCIC (Jiroux et al. 2019). However, none has focused on the pollination function, apart from bees (Menegus 2018). Our study did not aim to provide an exhaustive inventory of the entomofauna, but rather an ecosystemic approach. We chose to characterise plant–pollinator interactions by capturing insects regularly visiting wildflowers along transects and static observations, as pan traps do not reflect these interactions (O'Connor et al. 2019).

This paper aims to: (1) make public the data of insect flower visitors sampled in a thermo-Mediterranean scrubland maquis over 13 months spread over 2 years, (2) show the differences of floral visitor communities in spring between two consecutive years and (3) study the dynamic of floral insect corteges throughout a year.

## General description

### Purpose

Our aim is to publish in open access the records of insect visiting flowers collected during a 13-months study on plant-pollinator interactions in Corsica.

## Project description

### Title

Insect floral visitors of thermo-Mediterranean shrubland maquis (Ajaccio, Corsica, France).

### Personnel

Pierre-Yves Maestracci; Laurent Plume; Marc Gibernau and students.

### Study area description

Sampling was conducted on three sites near Ajaccio namely Loretto, Suartello and Vignola (Table 1) representing the ecological compensation zones for the Loregaz project and managed on its behalf by an association, the Conservatoire d'Espaces Naturels de Corse. On each site, the main vegetation is the Mediterranean maquis and the sampling design took into account the environmental differences within and amongst sites in order to have a good vegetation representation.

### Design description

The data published in this paper are part of a larger research project including plant-pollinator insect interaction networks (Nicolson and Wright 2017) and their dynamics over time (Burkle and Alarcón 2011).

### Funding

UMR SPE 6134, CPER project N°40137 “BiodivCorse – Explorer la biodiversité de la Corse” (Collectivité de Corse – Ministère de la Cohésion du territoire et des Relations avec les Collectivités territoriales), Lab. CRIGEN-ENGIE and CIFRE doctoral programme (ENGIE/Lab. CRIGEN-Univ. Corsica-Univ. Panthéon-Assas), ENGIE GPL for 2021 preliminary study.

## Sampling methods

### Sampling description

On each of the three sites every two weeks from March to May 2021 and every two weeks from mid-February to mid-November 2022, all insects visiting flowers were collected during the different time slots of the day : Morning (9 h-12 h), mid-day (12 h-14 h) and afternoon (14 h-17 h). For each time slot, two pollinating insect sampling methods were carried out consecutively at the three study sites (Loretto, Suartello and Vignola). The first method was dynamic and all the insects visiting flowers were collected along two transects (30 m long and 2 m wide) for 30 min/transect. The transects crossed the different types of vegetation in the studied area. The second method was static and consisted in capturing all the insects visiting the flowers for a period of 5 minutes on two different plants of the same species. For each field session, six different characteristic flowering species were selected depending on their abundance in the environment, resulting in a total of 12 flowers observed during a total period of 1 hour. The selected six species changed throughout the year according to their flowering seasons (Table 2 and Suppl. material 1).

The sampling consisted of three sampling protocols per site: 2 dynamic sessions + 1 static session (1^st^ week), 1 dynamic session + 2 static sessions (2^nd^ week) and 1 dynamic session + 1 static session (3^rd^ week). This sequence was repeated during all the sampling period.

In total, over the three sites in 2021 (Table 1), 54 transects (equivalent to 27 hours) were sampled with the dynamic method and 24 flowers observations were achieved using the static method (equivalent to 24 hours). In 2022, 172 transects (equivalent to 86 hours) were sampled with the dynamic method and 77 flowers observations were achieved using the static method (equivalent to 77 hours) (Table 1).

These two methods were chosen because of the complementary information of the dynamic and static sampling in order to obtain a better representation of the floral visitor insect communities (Table 3).

Inter-annual abundance and species diversity were compared using a Chi-square test and pairwise comparisons took into account Bonferroni statistical correction obtained with Past 4.14 statistical software (Hammer et al. 2001). Interannual comparision are made for the same months (March-April) to compare what is comparable.

## Geographic coverage

### Description

**Figure 1.**
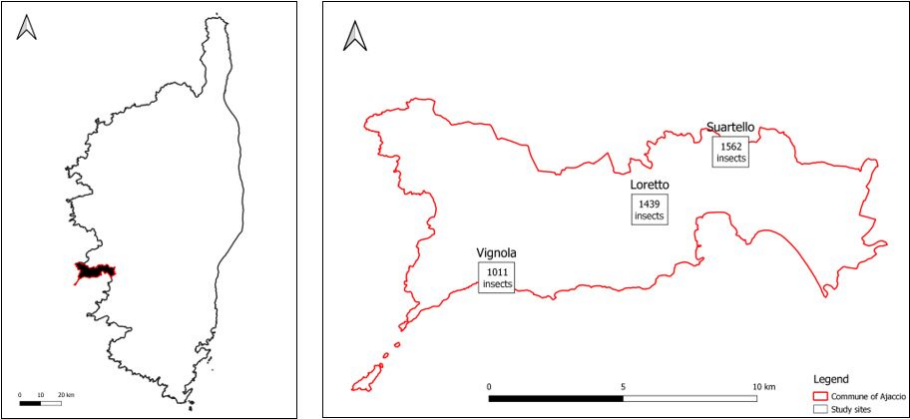
Geographical localisation of the three studied sites and total specimen abundances sampled per site.

**Figure 2.**
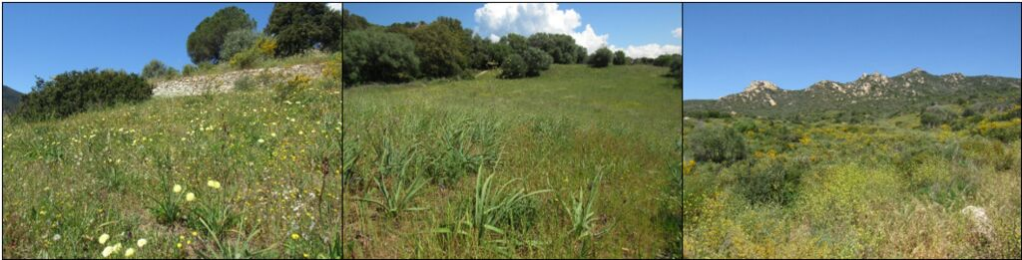
Floral habitats of the three sites (Loretto on the left, Suartello in the middle and Vignola on the right).

South-west Corsica, Ajaccio Region (Fig. 1): The Loretto site, located a few hundred metres from the city centre of Ajaccio adjoining the industrial Loregaz site, is made up of a plant mosaic, alternating open areas and groves (Table 4 and Fig. 2). The Suartello site, located on the edge of a wooded area, is made up of an open environment (e.g. grassland) and a plant mosaic environment (Table 4 and Fig. 2). The Vignola site facing the sea (ca. 200 m inland) was partly degraded by heavy rotary grinding in 2018, 4 years before the study. The proximity of the sites to each other makes it possible to consider their average temperatures and precipitation as being similar. Thus, they have a warm temperate climate with an average annual temperature of 16.3°C. However, some differences exist; Vignola is more exposed to sea spray and Suartello is slightly shadier due to the presence of trees on one side (Table 4 and Fig. 2).

## Taxonomic coverage

### Description

4012 specimens were sampled. A total of 252 species or morpho-species are identified in the collection (Suppl. material 1). The specimens belong to the orders Hymenoptera [1368 specimens], Coleoptera [2187 specimens], Diptera [288 specimens] and Lepidoptera [152 specimens]. Amongst these orders, we distinguish particularly the following families (Table 5):

- Order Hymenoptera: Apidae [720], Colletidae [149], Megachilidae [146], Halictidae [112], Andrenidae [108], Vespidae [42], Philanthidae[16], Sphecidae [12], Scoliidae [10].
- Order Coleoptera: Melyridae [448], Scarabaeidae [417], Mordellidae [384], Oedemeridae [300], Chrysomelidae [298], Nitidulidae [128] Buprestidae [108], Cerambycidae [41], Meloidae [28], Dermestidae [17].
- Order Diptera: Syrphidae [139], Bombyliidae [75], Muscidae [22], Rhiniidae [10].
- Order Lepidoptera: Lycaenidae [93], Nymphalidae [25], Pieridae [16].

Families with less than 10 specimens are grouped in Other Hymenoptera [37], Other Coleoptera [7], Other Diptera [17] and Other Lepidoptera [17].

The specimens identified only up to the order are included in the database: Diptera [25], Hymenoptera [16], Coleoptera [11] and Lepidoptera [1].

Insects identified in other orders, Hemiptera [13] or Dermaptera [4], incidentally sampled, are also included in the database.

In total, 133 genera have been identified, but only six were represented by more than 200 specimens, namely: genera *Apis*, *Bombus*, *Psilothrix*, *Mordellistena*, *Oedemera* and *Tropinota* (Table 6).

### Taxa included

**Table:** 

Rank	Scientific Name	Common Name
kingdom	Animalia	Animals
phylum	Arthropoda	
class	Insecta	Insects
order	Coleoptera	
order	Diptera	
order	Lepidoptera	
order	Hymenoptera	
superfamily	Chalcidoidae	
family	Andrenidae	
family	Anthomyiidae	
family	Apidae	
family	Bombyliidae	
family	Braconidae	
family	Brentidae	
family	Buprestidae	
family	Carabidae	
family	Cerambycidae	
family	Chalcididae	
family	Chrysididae	
family	Chrysomelidae	
family	Coccinellidae	
family	Colletidae	
family	Conopidae	
family	Crabronidae	
family	Curculionidae	
family	Dermestidae	
family	Empididae	
family	Formicidae	
family	Gasteruptionidae	
family	Halictidae	
family	Hesperidae	
family	Ichneumonidae	
family	Lycaenidae	
family	Megachilidae	
family	Meloidae	
family	Melyridae	
family	Mordellidae	
family	Muscidae	
family	Nitidulidae	
family	Nymphalidae	
family	Oedemeridae	
family	Papilionidae	
family	Philanthidae	
family	Pieridae	
family	Rhagionidae	
family	Rhiniidae	
family	Scarabaeidae	
family	Scoliidae	
family	Sesiidae	
family	Sphecidae	
family	Sphingidae	
family	Stratiomyidae	
family	Syrphidae	
family	Tachinidae	
family	Vespidae	

## Temporal coverage

**Data range:** 2021-3-02 – 2022-11-15.

### Notes

Specimens were collected over several months in 2021 (from March to May) and 2022 (from February to November).

## Collection data

### Collection name

SPE_Insects_Collection

### Specimen preservation method

Dried and pinned specimens and specimens in 70° alcohol.

## Usage licence

### Usage licence

Creative Commons Public Domain Waiver (CC-Zero)

## Data resources

### Data package title

Insect floral visitors of thermo-Mediterranean shrubland maquis (Ajaccio, Corsica, France)

### Resource link

https://doi.org/10.5281/zenodo.10781143

### Number of data sets

1

### Data set 1.

#### Data set name

Insect_floral_visitors_data_Corsica_France.csv

#### Data format

CSV UTF-8 (tab delimited values)

#### Download URL

https://doi.org/10.5281/zenodo.10781143

#### Data format version

Darwin core

#### Description

The whole dataset includes 4012 specimens from Ajaccio Region, south-west Corsica. This dataset includes our own identifications of the authors with geo-localisation within Corsica, France.

**Data set 1.** 

Column label	Column description
occurrenceID	Individual identification: combination of Museum name, collection identification, box number and specimen number within each box.
basisOfRecord	The specific nature of the data record (i.e. PreservedSpecimen).
eventDate	Event date in format YYYY-MM for 2022, in format YYYY-MM-DD for 2021.
year	Year of capture if known.
month	Month of capture if known.
day	Day of capture if known.
verbatimEventDate	Date of capture, in format YYYY-MM for 2022, in format YYYY-MM-DD for 2021.
scientific name	Lowest taxonomic rank possible, usually the species name. If the species is unknown, the genus or family names are given.
kingdom	Kingdom (i.e. Animalia).
phylum	Phylum (i.e. Arthropoda).
class	Class (i.e. Insecta).
order	Order.
family	Family name.
genus	Genus name.
specificEpithet	Species epithet of the scientificName.
infraspecificEpithet	Infra-specific epithet of the scientificName (subspecies).
taxonRank	Taxonomic rank of the most specific name in the scientificName.
identifiedBy	Name of the entomologist who identified the specimen, if indicated by the label.
dateIdentified	Year of identification, if known.
decimalLatitude	Geographic latitude (in decimal degrees) of the location.
decimalLongitude	Geographic longitude (in decimal degrees) of the location.
geodeticDatum	Coordinate system and set of reference points upon which the geographic coordinates are based (i.e. WGS 84).
country	Country of capture (France)
countryCode	Two letter country code of the specimen origin (FR).
locality	Location of capture, usually the locality (3 locality: Loretto, Suartello and Vignola).
stateProvince	French departmental administrative division (Corse-Du-Sud).
municipality	French municipality (Ajaccio)
institutionCode	Place where the specimen is held (University of Corsica - CRIGEN-ENGIE).
catalogNumber	Box identifier.
organismQuantity	Number of individuals bearing the same label (usually 1).
organismQuantityType	Individuals.
previousIdentifications	Species name originally given by the original collector, if different from scientificName.
coordinateUncertaintyInMeters	Uncertainty in coordinates (a few hundred metres at most).
georeferencedBy	Identity of the person who added the Latitude and longitude data, usually Maestracci Pierre-Yves.
georeferenceProtocol	How the georeference was computed, i.e. from label data (Locality).
georeferenceSources	Georeference code was inferred from geoportail.fr.
georeferencedDate	Georeference work was performed in 2023.
language	French and English.
collectionCode	Code of the collection (InsectsPollinators).
recordedBy	Name of collector.
identificationVerificationStatus	Usually 0.

## Additional information

**Specimen identification**

Morphological identifications (Hymenoptera and Lepidoptera: P-Y Maestracci and A. Cornuel-Willermoz, Diptera and Coleoptera: L Plume, Syrphidae: V. Sarthou and T. Lebard) and several CO1 barcoding (unpub. data).

Morphological identifications were possible thanks to reference works (Albouy and Richard 2017, SAPOLL 2018, Jiroux et al. 2019, Michez et al. 2019, Rasmont et al. 2021, Sarthou and Sarthou 2021, Cooper et al. 2022) and checklists (Wiemers et al. 2018, Ghisbain et al. 2023).

**Contacts**

University of Corsica: maestracci_p@univ-corse.fr and gibernau_m@univ-corse.fr

**Dataset management**:

UnivCorse: maestracci_p@univ-corse.fr

### General Discussion

**Global abundance & Diversity**

**Figure 3.**
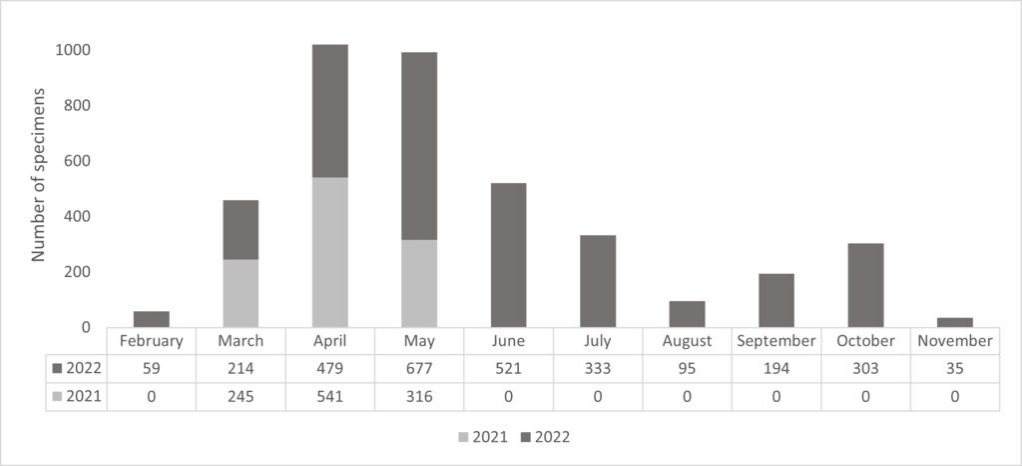
Monthly variation of sampled specimens in 2021 and 2022.

Over the 13 months of the study spread over 2 years, a total of 4012 specimens were sampled, high numbers of specimens were obtained in spring (March-June) and in September-October (Fig. 3).

Our database represents a total of 252 insect species and morpho-species (Suppl. material 1). The richest insect order was the Hymenoptera with 39.4% of the morpho-species, followed by the Diptera (27.2%), the Coleoptera (24.4%) and, finally, the Lepidoptera (8.7%). On the other hand, the Coleoptera was the most abundant insect order representing 54.5% of the sampled individuals, with notably two species *Psilothrixviridicoerulea* (403 insects) and *Tropinotasqualida* (282 insects). The Hymenoptera was the second most abundant insect order representing 34.1% of the specimens and the two most represented species were *Apismellifera* (318 insects) and *Bombusxanthopus* (214 insects). Lastly, the Diptera (7.2%) and the Lepidoptera (3.8%) were the less abundant orders in our sampling.

**Site specificities**

When calculating the sampling completeness for the three sites, the diversity of Suartello (0.72) and Loretto (0.80) appeared to have been better sampled than from Vignola with a completeness of only 0.59. Consequently, the estimate of total species diversity (Table 7) was higher in Vignola (245.6 by Chao1 or 238.6 by ACE) than in Loretto (201.6 by Chao1 or 203.5 by ACE) or Suartello (225.9 by Chao1 or 214.4 by ACE). Such result is partly due to a relatively higher percentage of singletons (species sampled only once) in Vignola (43.7% of the 144 species) than in Loretto (30.4% of the 161 species) or Suartello (33.3% of the 162 species).

When looking at the site differences in terms of species composition (Table 8 and Table 9), the site of Vignola appeared to be slightly different from the other two sites (Loretto and Suartello). Such diversity difference could be due to the geographical distance, the coastal location (Fig. 1) and/or the specificity of the site in terms of habitat and vegetation (Table 1).

**Annual variation 2021-2022**

**Figure 4.**
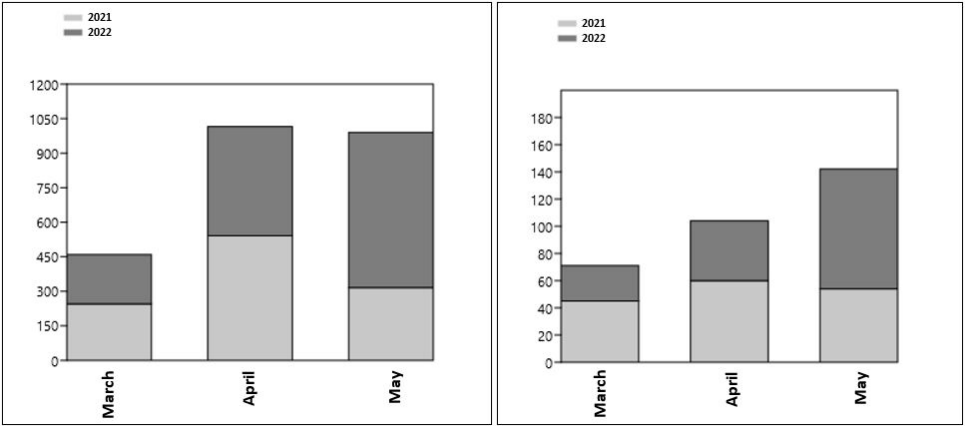
Inter-annual variation of insect abundance (left graph) and species diversity (right graph) over 3 months between 2021 and 2022 (Past 4.14 statistical software, Hammer et al. (2001)).

Globally, our sampling of the floral visitors on the three studied sites in 2021 coincided with 3 months (March, April and May) of our survey of 2022 (Fig. 3 and Fig. 4). Taking into account that the sampling effort in May 2021 was half of that of 2022, we statistically compare inter-annual variation only for the months of March and April (composed for March 2021: 14 dynamic sessions and eight static sessions; for April 2021: seven dynamics and 13 statics; for March 2022: eight dynamics and eight statics; and for April 2022: six dynamics and nine statics). Thus, there were no statistical differences for both abundance and species diversity (Chi-square tests, p > 0.45). For May, the higher abundance observed in 2022 (n = 674) is about twice the abundance found in May 2021 (n = 316) and the species diversity showed similar trends (54 species in 2021 and 88 species in 2022); such results were probably due to the difference in the sampling effort.

The monthly insect abundance per order significantly varied between the two years (Chi² = 136.24, df = 9, p < 10^-6^). Significant variations were detected for Hymenoptera (p = 1.5 x 10^-3^) and for Coleoptera (p = 4.5 x 10^-3^) amongst the four sampling periods, indicating both monthly and yearly differences. On the other hand, no statistically differences were detected for Diptera and Lepidoptera. The monthly species diversity per order did not significantly vary between the two years (Chi² = 10.68, df = 9, p = 0.3). No species diversity variation was detected for the four orders.

**Monthly annual variation in 2022**

**Figure 5.**
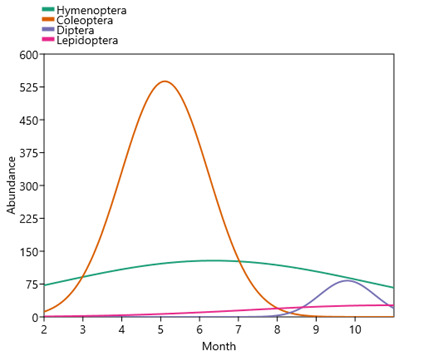
Insects’ abundance sampled according to orders and months (module Species packing Gaussian, Past 4.14 statistical software, Hammer et al. (2001)).

In 2022, insects visiting flowers were sampled during 10 successive months (Fig. 3). Interestingly, the annual distribution of specimens was not homogenous and varied amongst insect orders (Fig. 5). Coleoptera are mainly present in spring (April, May and June) representing 82.7% of the sampled beetles (1257 insects). Hymenoptera appeared to be present evenly all year round. Diptera are mainly active on flowers at the end of summer (September and October) with 56.7% of captured flies (123 insects). Finally, Lepidoptera were rare in our sampling (maximum of 26 specimens during a given month), but their number appeared to linearly increase between spring and autumn (Fig. 5).

**Figure 6.**
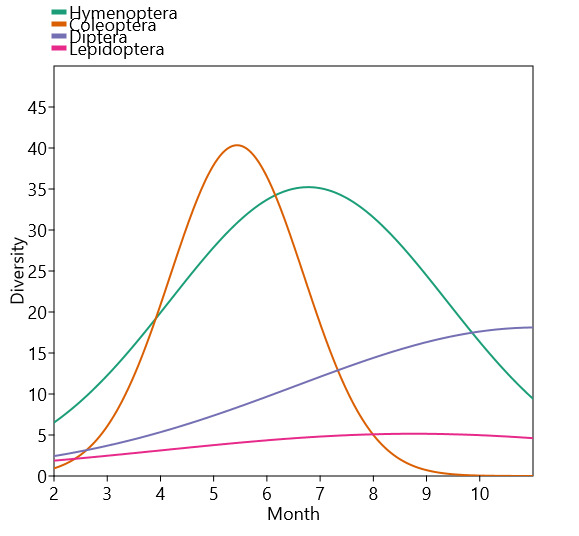
Insects’ diversity sampled according to orders and months (module Species packing Gaussian, Past 4.14 statistical software, Hammer et al. (2001)).

In terms of species diversity per insect order, slightly different results were obtained (Fig. 6). For Coleoptera, as for the abundance, the species diversity occurred mainly during the late spring (May and June) with 88.3% of the Coleoptera diversity sampled during these two months which represents 23.7% of total species diversity. On the other hand, the species diversity of Hymenoptera was higher in summer (June and July and August) with 63.3% of Hymenoptera diversity sampled during these three months, representing 25.4% of total species diversity. The species diversity of Diptera presented a different pattern being low at the beginning of the year (February) and regularly increasing during the year until reaching a maximum in October. In fact, 65.4% of the species diversity of Diptera were captured in September and October, representing 16.1% of the total species diversity. Finally, the species diversity of Lepidoptera is relatively low (maximum four species) and quite regular through the year (Fig. 6).

### Conclusion

In our data, the diversity of orders of flower-visiting insects and their relative abundance are not linked. Beetles are by far the most abundant with more than half of individuals belonging to this order. Howewer, they are not the most diverse since a third of the species belonged to the Hymenoptera order.

By considering the entire year rather than a limited period as is generally the case in other studies, we consider to have obtained a better representation of the Mediterranean insect community visiting flowers with an almost exclusive presence of Coleoptera in spring and early summer and Hymenoptera, Diptera and Lepidoptera until late in the year. Indeed, the climate of Corsica and, more specifically, the coastal climate, allows late flowering of plant species and, therefore, a late period of activity for the associated insects. In addition, the observed inter-annual variations of these flower-visiting insects, both for the abundances and the species diversities, suggest that these insect communities are highly dynamic.

The insects visiting flowers represent an important proportion of the insect diversity and focusing on these communities is interesting for understanding their complex insect-plant interactions at the ecosystem level. Our next work will focus on establishing the pollination efficiency of these different flower-visitor insects and further studying these plant-insect interaction networks.

## Supplementary Material

A97AB9C7-C008-5469-A8E3-2D9EA467FC32 10.3897/BDJ.12.e118614.suppl1 Supplementary material 1 List of Species Data type Species list Brief description List of Species of Insect floral visitors of thermo-Mediterranean shrubland maquis (Ajaccio, Corsica, France), including plant and insects. File: oo_1021513.xlsx https://binary.pensoft.net/file/1021513 Pierre-Yves Maestracci; Marc Gibernau; Laurent Plume

## Figures and Tables

**Table 1.** Number of transects and flower observations and their hour equivalents per studied sites.

**Site**	**2021**		**2022**	
	**Dynamic sessions**	**Static sessions**	**Dynamic sessions**	**Static sessions**
Loretto	9	8	28	26
Suartello	9	8	28	26
Vignola	9	8	30	25
Total (h)	27	24	86	77

**Table 2.** Plant species of the static method chosen according their phenology.

**Scientific name**	**Period**
Anthemis arvensis L., 1753	Summer
Asphodelus ramosus L., 1753	Spring
Bunias erucago L., 1753	Spring
Calendula arvensis L., 1763	Spring
Carduus pycnocephalus L., 1763	Summer
Carlina corymbosa L., 1753	Summer
Chondrilla juncea L., 1753	Summer
Cistus creticus L., 1759	Spring
Cistusmonspeliensis L., 1753	Spring
Cistus salviifolius L., 1753	Spring
Cytisuslaniger (Desf.) DC., 1805	Spring
Daphne gnidium L., 1753	Summer
Daucus carota L., 1753	Summer
Dittrichia viscosa (L.) Greuter, 1973	Summer
Echium plantagineum L., 1771	Spring
Erica arborea L., 1753	Spring
Eryngium campestre L., 1753	Summer
Foeniculum vulgare Mill., 1768	Summer
Fumaria capreolata L., 1753	Spring
Glebionis segetum (L.) Fourr., 1869	Summer
Helichrysum italicum (Roth) G.Don, 1830	Summer
Heliotropium europaeum L., 1753	Autumn
Hypericum perforatum L., 1753	Spring
Knautia integrifolia (L.) Bertol., 1836	Spring
Lavandula stoechas L., 1753	Spring
Leontodon tuberosus L., 1753	Autumn
Lupinus angustifolius L., 1753	Spring
Myrtus communis L., 1753	Summer
Phillyrea angustifolia L., 1753	Spring
Raphanus raphanistrum L., 1753	Spring
Reichardia picroides (L.) Roth, 1787	Spring
Smilax aspera L., 1753	Autumn
Tolpis virgata Bertol., 1803	Summer
Urospermum dalechampii (L.) Scop. ex F.W.Schmidt, 1795	Spring
Verbascum sinuatum L., 1753	Summer
Vicia villosa Roth, 1793	Spring

**Table 3.** Abundance and diversity of insect pollinators according to the two sampling methods.

	**Dynamic method**		**Static method**		**Total**
**Year**	2021	2022	2021	2022	
Abundance	683	1747	419	1163	4012
Diversity	82	191	49	164	252

**Table 4.** Studied sites and detailed main characteristics (geographical and vegetation).

Locality	Geographical coordinates		Orientation	Main Vegetation	Area (ha)
	Decimal latitude and longitude	Altitude (m)			
Loretto	41.933698, 8.718367	85	S	Wasteland [CORINE-Biotope: 87.1); Matorral with olive trees and mastic trees [CORINE-Biotope: 32.12)	1.9
Suartello	41.953102, 8.755813	90	SSE	Grassland [CORINE-Biotope: 34.4]; High maquis of the western Mediterranean [CORINE-Biotope: 32.311]	2.5
Vignola	41.912298, 8.650145	30	SW	Medium maquis with *Cytisuslaniger* and *Pistacialentiscus* in mosaic with *Oleaeuropea* – Fruity calicotome [CORINE-Biotope: 32.215]; Maquis with *Cistusmonspeliensis* [CORINE-Biotope: 32.341]	18

**Table 5.** List of taxa (n > 10 specimens) included in the database.

**Rank**	**Scientific name**
Order	Hymenoptera
family	Andrenidae
family	Apidae
family	Colletidae
family	Halictidae
family	Megachilidae
family	Philanthidae
family	Scoliidae
family	Sphecidae
family	Vespidae
Order	Coleoptera
family	Buprestidae
family	Cerambycidae
family	Chrysomelidae
family	Dermestidae
family	Meloidae
family	Melyridae
family	Mordellidae
family	Nitidulidae
family	Oedemeridae
family	Scarabaeidae
Order	Diptera
family	Bombyliidae
family	Muscidae
family	Rhiniidae
family	Syrphidae
Order	Lepidoptera
family	Lycaenidae
family	Nymphalidae
family	Pieridae

**Table 6.** Genera with more than 200 specimens and the corresponding numbers of species per genus.

**Genus**	**Number of specimens**	**Number of species or morpho-species identified in the sample**
*Apis*	318	1
*Bombus*	244	6
*Psilothrix*	403	1
*Mordellistena*	384	10
*Oedemera*	300	8
*Tropinota*	282	1

**Table 7.** Diversity indices (number of species and specimens, Shannon index and the estimate number of species with the improved Chao1 estimator or the Abundance-base Coverage Estimator) for the three sites obtained with Past 4.14 statistical software (Hammer et al. 2001).

	Loretto	Suartello	Vignola
Taxa_S	161	162	144
N	1433	1560	1005
Shannon	4.035	3.983	3.933
iChao1	201.6	225.9	245.6
ACE	203.5	214.4	238.6

**Table 8.** Beta diversity (Whittaker) comparisons amongst the studied three sites (Past 4.14 statistical software, Hammer et al. (2001)).

	Loretto	Suartello	Vignola
Loretto	0	0.34365	0.37705
Suartello	0.34365	0	0.4183
Vignola	0.37705	0.4183	0

**Table 9.** Jacard similarity indices amongst the three sites studied (Past 4.14 statistical software, Hammer et al. (2001)).

	Loretto	Suartello	Vignola
Loretto	1	0.48847926	0.45238095
Suartello	0.48847926	1	0.41013825
Vignola	0.45238095	0.41013825	1
